# Understanding the link between gut microbiota, dietary intake, and nutritional status in children with autism and typical development

**DOI:** 10.3389/fnut.2023.1202948

**Published:** 2023-07-20

**Authors:** Paula Mendive Dubourdieu, Marcela Guerendiain

**Affiliations:** Área de Investigación, Escuela de Nutrición, Universidad de la República, Montevideo, Uruguay

**Keywords:** autism spectrum disorder, gut microbiota, nutritional status, gluten- and casein-free diet, dietary intake, children, adolescents

## Abstract

**Background:**

Gut microbiota plays a potential role in human health and different disorders such as autism spectrum disorder (ASD). Therefore, we analyzed gut bacteria composition in children with ASD and typical development (TD), and its relationship with nutritional status and dietary intake.

**Methods:**

A descriptive cross-sectional study was carried out in 3- to 12-year-old children (ASD = 30, TD = 28). Dietary intake (applying food frequency questionnaires) and body mass index-for-age (expressed in z-score) were determined. Children were divided into normal weight and excess weight (risk of overweight + overweight + obesity), and the ASD group was categorized into gluten- and casein-free diet (ASD-diet) or no diet (ASD-no diet). The relative abundance of gut bacteria was analyzed in fecal samples by 16S rRNA sequencing.

**Results:**

Children with excess weight had lower *Roseburia* than normal weight. Fewer *Bifidobacterium longum* and higher *Clostridium glycolicum* were found in the ASD group compared with TD one. Participants with excess weight and ASD had lower *Roseburia* and *Faecalibacterium prausnitzii* and higher *Eubacterium ventricosum* and *Flavonifractor plautii* than the TD group with the same nutritional status. Positive and negative associations were found between the bacteria genus and species, and the intake of dairy, vegetable drinks, cereals with and without gluten, food source of proteins, fish, food source of fat, and coconut oil, in unadjusted models and after adjustment for age, diet/no diet, ASD/TD.

**Conclusion:**

Significant differences in microbial community composition were found between children with ASD and TD, considering their nutritional status and dietary intake.

## Introduction

Autism spectrum disorder (ASD) is a neurodevelopment condition that has had a rapidly increasing prevalence. However, there is no standard treatment due to its complex ethology, involving genetic and environmental factors (1, 2). In the last few decades, it has been recognized that gut microbiota plays a major role in human health and different disorders such as autism (3). Multiple cohort studies indicate that several inflammation-related disorders and neurodevelopmental diseases have been associated with alterations in the gut ecosystem, a condition known as dysbiosis (4, 5). For example, a greater relative abundance of certain bacteria such as *Clostridium* and *Sutterella* has been observed in children with ASD as opposed to typical development (TD) ones, but the findings from different investigations are still controversial (6).

Robust literature data show that there is a two-way communication between gut and the brain, in which microbiota, the enteric nervous system, autonomic nervous system, endocrine system, immune system, and central nervous system are involved (4). Bacterial metabolites have been shown to be implicated in the secretion of neurotransmitters that are part of memory, learning, and behavioral processes (7). Diet plays an important role in gut microbiota homeostasis and metabolism, and children with ASD have difficulties in maintaining a balanced diet due to multiple factors such as highly selective food preference and gastrointestinal problems (8). In addition, many families with children with ASD have chosen to follow a gluten- and casein-free diet (GCFD) under the unproven hypothesis that these proteins are metabolized into gluteomorphin and casomorphin and that, via a leaky gut, they bind to opiate receptors in the central nervous system causing autism symptoms (9). There is still no consensus on the use and effectiveness of this type of diet for treating autism, and additional studies are needed to describe the effects of the diet on gut microbiota (9–11).

Dietary intake can modulate gut microbiota throughout life, and this action would depend on the type and amount of foods chosen, which can inflect up to 60% of the microbiome composition since it provides countless substrates for microbial metabolism (12, 13). Furthermore, there are bacteria with specific enzymes that convert certain nutrients into different metabolites that influence brain function (14). For example, from the metabolism of tryptophan, it is possible to obtain indole which has a positive effect on mental health, but another metabolite such as indoxyl sulfate has been linked to the development of ASD.

In relation to food intake and its association with bacterial taxonomy, a study of European children (fed with a Western diet rich in animal protein) and African children (fed with local vegetables and whole grains) showed that children in Burkina Faso have higher levels of *Prevotella* and lower levels of *Bacteroides* and *Enterobacteriaceae* than children from Italy (15). This diet and intestinal microbiota in rural African children have been linked to lower inflammatory conditions and infectious colonic diseases (11, 15). On the contrary, it has been shown that diets with a high intake of red meat, refined carbohydrates and fat, and a lower consumption of fish and vegetables could cause dysbiosis (13).

Moreover, scientific evidence has shown that nutritional status is related to gut microbiota (16). There is a lack of consensus as regards a healthy-type taxonomic microbiome composition, but recent studies show that there is a difference in gut bacteria between obese and lean children and adolescents (17). Bervoets et al. (18) observed that children with obesity had a higher level of *Lactobacillus* spp. and a lower level of *Bifidobacterium vulgatus* than the lean ones. A prospective study showed that obesity in children was associated with an increase in *Bacteroidaceae* and a lower relative abundance of *Prevotellaceae* compared with children with normal weight (16). The relation between gut bacteria and weight gain is still unclear (12).

It has been shown that early intervention in children's gut microbiota can help prevent health disorders, but it is necessary to elucidate the link between diet and intestinal microbe composition to define a strategy to improve their health (19, 20). The symptoms and comorbidities of ASD could be improved with dietary interventions carried out after a deeper understanding of how foods relate to the intestinal microbiota (14). Therefore, the purpose of this study was to analyze gut bacteria composition in children with ASD and TD, and its relationship with nutritional status and dietary intake.

## Materials and methods

### Participants and ethics statement

**Figure 1:**
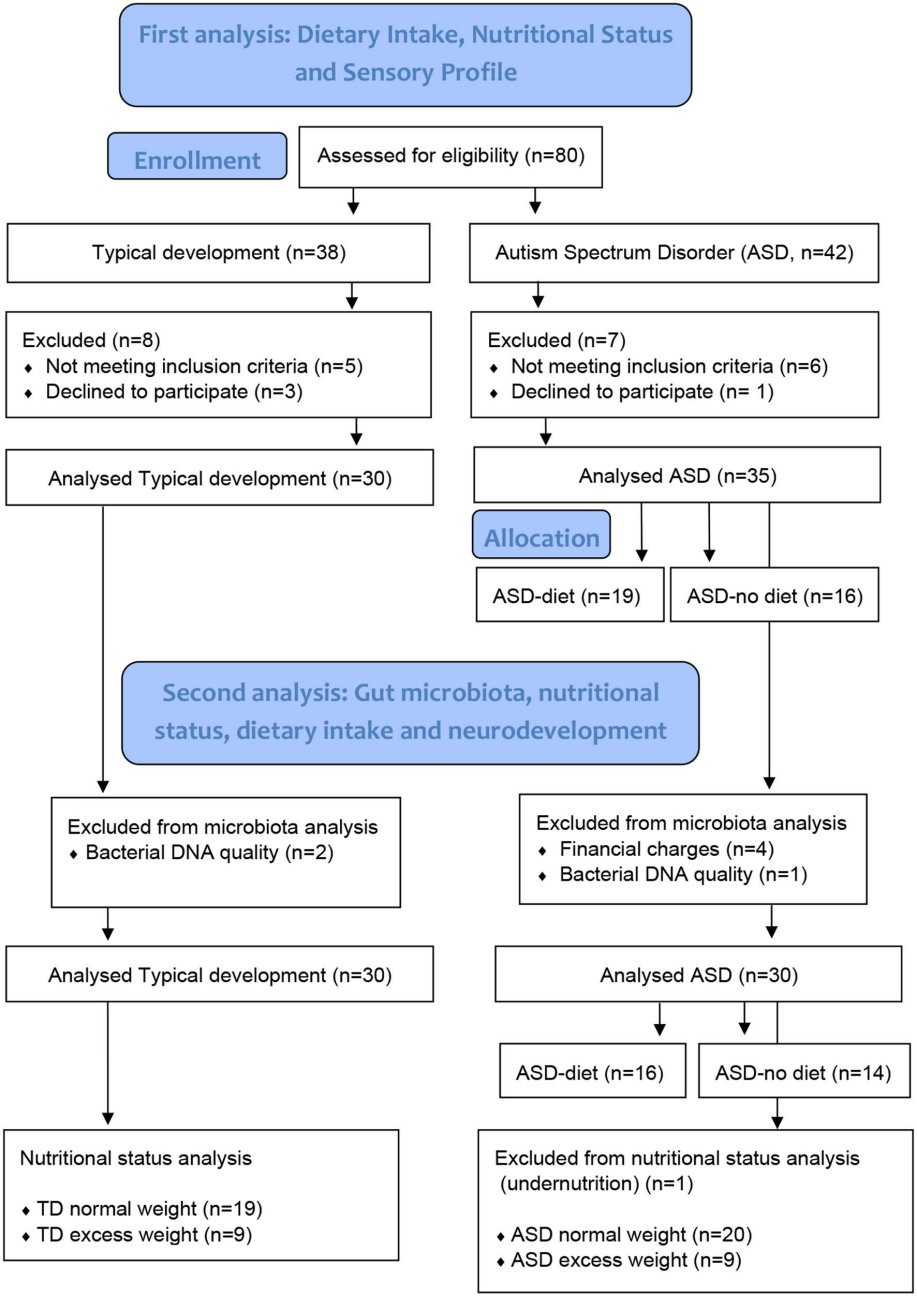
Participants' flow chart.

From February to March 2020, we recruited a total of 65 children and adolescents aged 3 to 12 years at the nutritionist's office in Montevideo, Uruguay, through an open call (8); in this gut microbiota study, 30 with ASD and 28 neurotypicals were included (Figure 1). Diagnoses of ASD by a psychiatrist or a pediatric neurology specialist met the criteria of the Diagnostic and Statistical Manual of Mental Disorders (DSM-V) (21). Participants had not been taking medication, antibiotics, or probiotics for at least 1 month prior to enrollment in the study, and no children in the TD group were on a restricted diet. In addition, those diagnosed with attention deficit and hyperactivity disorder, diabetes mellitus, genetic diseases, inborn errors of metabolism, inflammatory bowel disease, celiac disease, and motor disability were excluded from both groups. This research was performed in accordance with the Helsinki Declaration 2000, approved by the Research Ethics Committee of the School of Nutrition, University of the Republic, and registered with the Ministry of Health of Uruguay (No. 282599). The study was explained to the participants' parents by telephone and discussed personally during their first visit to the research clinic, where informed written consent was obtained from every parent.

### Sample collection, gut microbiota sequencing, and taxonomic classification

Children's parents were given a fecal microbiota kit (tube with transport media and specimen collection swab) and thorough instructions as to how they should collect the stool samples from their children at home. They collected a single fecal sample that was refrigerated until delivery to the clinic within 48 h. Once received, the samples were transferred to the laboratory with a cold pack and stored in an ultra-freezer at −80°C until analysis.

Extraction of bacterial DNA was performed at Enteria SRL laboratory, which followed the protocol recommended by Quick–DNA Fecal/Soil Microbe Miniprep Kit (Zymo Research—Catalog No. D6010). The quantity and quality of DNA were assessed by measuring absorbance at 260 and 280 nm using the Tecan Infinite M200 Pro (absorbance range 1.8–2 OD280/260). The extracted genomic DNA was sent to Genia laboratory to amplify hypervariable V1-V9 regions of the 16S rDNA gene from bacteria with Ion 16S™ Metagenomics Kit in PCR cycler using the Ion Torrent™ semiconductor sequencing workflow. Amplified fragments were sequenced using the Ion PGM™ Sequencing 400 Kit on the Ion PGM™ platform and analyzed using the Ion 16S™ metagenomics analysis module within Ion Reporter™ software.

Stool samples were studied to determine the relative abundance of gut bacteria. Operational taxonomic units (OTUs) were defined at 97% sequence homology, and the abundances of these bacterial genera or species were normalized. Taxonomic classification was performed using the SILVA 128 reference database up to the species level.

### Anthropometric measures and dietary intake

All protocols including anthropometric measures and dietary intake were performed as previously described by Mendive Dubourdieu et al. (8). Participants' height and weight were measured by the same nutritionist researcher. Height was determined by using a portable height rod (208 Seca) with a 810–2,060 mm range and a 1 mm precision, and weight was measured using a portable electronic scale (Seca 813, Hamburg, Germany) with a 100 g accuracy, while subjects were barefoot and wearing light clothing according to techniques standardized by Frisancho (22) and the World Health Organization (WHO) (22, 23). Data were analyzed in Anthro (for children aged 3 to 5 years) and Anthro plus (for children and adolescents aged 5 to 12 years) software (WHO v.1.0.2. 2007), which apply WHO child growth curves (22). Body mass index-for-age (BMI/A), expressed in z-score (z), was the indicator studied to classify children into one of the following categories: normal weight (NW) and excess weight (EW, risk of overweight + overweight + obesity). Cutoff points used for children aged 2–5 years were >3SD, obesity; >2SD, overweight; >1SD, risk of overweight; between <1SD and >-1SD, normal weight; ≤-1SD, risk of wasting; ≤-2SD, emaciation; and ≤-3SD, severe emaciation and in those over 5 years old were ≥2SD, obesity; ≥1SD, overweight; between <1SD and >-2SD, normal weight; ≤-2SD, wasting; and ≤-3SD, severe emaciation. Undernutrition was dismissed because the sample size was small, and therefore, those participants were not considered for anthropometric analysis.

Children with ASD were divided into two groups depending on whether they followed a GCFD (ASD-diet, *n* = 16) or did not have a restricted diet (ASD-no diet, *n* = 14). Food intake over the past 3 months was estimated based on data obtained through the SAYCARE study food frequency questionnaire (FFQ) (24), which was adapted to this study to gather information on the consumption of gluten-free and casein-free foods. Children's parents were asked to indicate the consumption frequency and portion size of each food item according to a food photo booklet as a reference. The average daily consumption of each food (g/day or ml/day) was calculated and organized into different groups as follows: (1) “dairy”: milk, yogurt, chocolate milk, dairy desserts, and cheese; (2) “vegetable drinks”: birdseed, chestnut, almond, oat, rice, and coconut drinks; (3) “cereals with gluten”: pasta, bread, cookies, bakery products, breakfast cereals, pizza, and *empanadas* (dough stuffed with meat, fish, vegetables, etc., baked or fried), “cereals without gluten”: the same foods in the previous group without gluten and rice; (4) “food source of proteins”: meat, minced meat, chicken, pork, eggs, fresh and canned fish, and *milanesa* steak with and without gluten (a thin slice of beef dipped in beaten eggs and breaded; the fact that 25% of its weight is due to cereal has been taken into account); (5) “food source of fat”: butter, ghee (fat obtained by heating cow milk butter), and oil. Dairy, cereals with gluten, cereals without gluten, food source of fat (for children with typical development and ASD without diet), vegetable drinks, and food source of proteins (for children with ASD and a GCFD) were divided into two subgroups, considering the 50th percentile of the intake (intake ≤p50 and >p50).

### Statistical analysis

For statistical analyses, IBM SPSS Statistics 22.0 (IBM Corp, Armonk, NY, USA) was used. We performed the Kolmogorov–Smirnov test to verify variable normal distribution. Data that were not normally distributed were log10 transformed. A *p*-value of lower than 0.05 was accepted as significant (two-tailed).

To compare genus and species relative abundance according to neurodevelopment (ASD vs. TD) and between autistic groups (ASD-diet vs. ASD-no diet), independent sample *t*-test was assessed. To study bacteria relative abundance according to nutritional status (NW and EW) in ASD and TD children, the two-way ANCOVA was applied (adjusted for age, birth weight, and GCFD/not restricted diet). Pair comparisons between the different groups were adjusted by the Bonferroni *post-hoc* test. Comparison between NW and EW in all children was carried out using one-way ANCOVA and correcting for the same potential confounders.

To evaluate associations between gut microbiota and dietary intake, univariate linear regression was used and adjusted for the following potential confounders: age, GCFD/not restricted diet, and TD/ASD (when all the children were analyzed); age (in the TD group), and age and GCFD/not restricted diet (in the ASD). In children with TD and ASD-no diet, differences in bacterial abundances among food intake p50 groups were examined using two-way ANOVA. Finally, the Student *t*-test and Mann–Whitney test were used to determine whether there were differences in gut bacteria relative abundances between those with higher or lower dairy intake in the ASD-diet group.

## Results

Anthropometric characteristics, dietary intake, and age of children with autism spectrum disorder and typical development have been previously published (8). In Table 1, we compared the mean relative abundance of 20 selected genera and 16 species, according to ASD-diet vs. ASD-no diet and ASD vs. TD groups. No significant differences were found in the relative abundances of bacterial genera between neurotypical and ASD children. However, there is a significant difference in the mean relative abundance of *Bifidobacterium* (*p* = 0.008), *Roseburia* (*p* = 0.002), and *Sutterella* (*p* = 0.015) between the ASD-diet and ASD-no diet groups. At the species level, the ASD-diet group showed fewer *Bifidobacterium adolescentis* (*p* = 0.046) and *Bifidobacterium longum* (*p* = 0.002) but higher *Roseburia hominis* (*p* = 0.002) than the ASD-no diet group. Additionally, the ASD group had lower *Bifidobacterium longum* (*p* = 0.002) and higher *Clostridium glycolicum* (*p* = 0.028) than TD children.

**Table 1:** Relative abundance of gut bacteria genus and species in children with autism spectrum disorder and typical development.

**Gut bacteria**	**ASD group**			**ASD group (*n =* 30)**	**TD group (*n =* 28)**	*p* ^**^
	**ASD-diet (*****n** =* **16)**	**ASD-no diet (*****n** =* **14)**	*p* ^*^			
**Genus**						
*Akkermansia*	1.39 ± 1.90	1.84 ± 2.74	0.618^a^	1.60 ± 2.30	1.45 ± 2.17	0.938^a^
*Alistipes*	3.61 ± 2.82	4.42 ± 3.61	0.494^b^	3.98 ± 3.18	4.30 ± 2.92	0.565^b^
*Bacteroides*	23.98 ± 11.66	21.36 ± 7.82	0.482^b^	22.75 ± 9.98	24.45 ± 10.14	0.554^b^
*Bifidobacterium*	1.44 ± 1.80	4.22 ± 3.62	**0.008** ^**a**^	2.73 ± 3.09	3.53 ± 2.35	0.054^a^
*Blautia*	2.84 ± 1.11	2.34 ± 1.18	0.114^a^	2.60 ± 1.15	2.65 ± 1.23	0.803^a^
*Clostridium*	6.47 ± 3.16	7.60 ± 3.65	0.280^a^	6.99 ± 3.39	6.90 ± 3.35	0.901^a^
*Coprococcus*	0.85 ± 0.84	0.63 ± 0.48	0.760^a^	0.74 ± 0.63	0.77 ± 0.69	0.962^a^
*Dialister*	0.60 ± 1.10	1.36 ± 1.65	0.105^a^	0.94 ± 1.40	0.67 ± 0.78	0.871^a^
*Enterococcus*	0.56 ± 2.03	0.10 ± 0.10	0.427^a^	0.34 ± 1.48	0.33 ± 0.83	0.122^a^
*Eubacterium*	3.66 ± 2.38	2.54 ± 1.54	0.114^a^	3.13 ± 2.08	3.07 ± 2.05	0.988^a^
*Faecalibacterium*	16.34 ± 7.68	11.72 ± 4.62	0.060^b^	14.18 ± 6.75	15.13 ± 5.09	0.312^b^
*Lachnoclostridium*	1.18 ± 0.81	0.96 ± 1.09	0.561^a^	1.07 ± 0.96	0.82 ± 0.71	0.586^a^
*Lactobacillus*	0.64 ± 0.65	1.03 ± 2.28	0.786^a^	0.82 ± 1.61	0.67 ± 1.26	0.697^a^
*Prevotella*	12.93 ± 12.52	10.87 ± 11.72	0.739^a^	11.96 ± 11.99	6.89 ± 9.16	0.093^a^
*Pseudomonas*	0.65 ± 2.46	0.05 ± 0.05	0.683^a^	0.37 ± 1.79	0.06 ± 0.06	0.534^a^
*Roseburia*	3.47 ± 1.98	1.32 ± 0.99	**0.002** ^**a**^	2.47 ± 1.92	2.89 ± 1.92	0.304^a^
*Ruminococcus*	2.55 ± 1.72	2.54 ± 1.23	0.618^a^	2.55 ± 1.48	2.81 ± 1.34	0.350^a^
*Streptococcus*	0.49 ± 0.33	2.27 ± 5.60	0.394^a^	1.32 ± 3.86	1.53 ± 1.71	0.058^a^
*Sutterella*	0.62 ± 1.22	2.13 ± 1.98	**0.015** ^**a**^	1.32 ± 1.77	1.78 ± 1.76	0.119^a^
*Turicibacter*	0.80 ± 1.21	0.62 ± 0.65	0.868^a^	0.72 ± 0.98	0.56 ± 0.60	0.749^a^
**Species**						
*Akkermansia muciniphila*	1.19 ± 1.40	2.27 ± 3.30	0.269^a^	1.69 ± 2.49	1.75 ± 2.60	0.935^a^
*Bacteroides frágilis*	0.45 ± 0.47	1.21 ± 2.85	0.301^a^	0.80 ± 1.97	1.21 ± 1.32	0.075^a^
*Bacteroides intestinalis*	0.02 ± 0.03	0.13 ± 0.33	0.944^a^	0.07 ± 0.23	0.55 ± 2.43	0.453^a^
*Bifidobacterium adolescentis*	0.30 ± 0.47	1.25 ± 2.10	**0.046** ^**a**^	0.74 ± 1.52	0.65 ± 1.12	0.463^a^
*Bifidobacterium longum*	0.23 ± 0.49	1.11 ± 2.67	**0.002** ^**a**^	0.64 ± 1.87	1.21 ± 1.80	**0.002** ^**a**^
*Clostridium bartletti*	0.42 ± 0.39	0.63 ± 0.63	0.647^a^	0.52 ± 0.09	0.22 ± 0.04	0.335^a^
*Clostriduim glycolicum*	0.42 ± 0.28	0.55 ± 0.49	0.678^a^	0.48 ± 0.39	0.31 ± 0.30	**0.028** ^**a**^
*Coprococcus comes*	0.19 ± 0.20	0.37 ± 0.36	0.156^a^	0.28 ± 0.29	0.27 ± 0.25	0.767^a^
*Eubacterium eligens*	1.76 ± 1.83	1.48 ± 1.53	0.561^a^	1.68 ± 0.30	1.01 ± 1.01	0.171^a^
*Eubacterium ventriosum*	10.77 ± 9.20	8.60 ± 8.14	0.618^a^	9.76 ± 8.64	12.58 ± 8.89	0.101^a^
*Faecalibacterium prausnitzii*	6.60 ± 11.80	3.95 ± 5.60	0.982^a^	5.36 ± 9.37	3.99 ± 7.69	0.735^a^
*Flavonifactor plautii*	0.95 ± 2.00	0.76 ± 1.03	0.802^a^	0.86 ± 1.60	0.89 ± 0.89	0.111^a^
*Lactobacillus reuteri*	0.29 ± 0.44	0.18 ± 0.42	0.498^a^	0.24 ± 0.43	0.30 ± 0.41	0.350^a^
*Lactobacillus salivarius*	0.04 ± 0.07	0.52 ± 1.71	0.386^a^	0.27 ± 1.17	0.03 ± 0.08	0.182^a^
*Roseburia hominis*	0.37 ± 0.40	0.10 ± 0.17	**0.002** ^**a**^	0.24 ± 0.34	0.49 ± 1.37	0.331^a^
*Trabulsiella odonototermitis*	0.63 ± 1.33	0.34 ± 0.55	0.483^a^	0.49 ± 1.03	0.50 ± 0.65	0.366^a^

The results are expressed as means ± SD. Statistically significant differences (indicated in bold): *p* < 0.05 (^a^Mann–Whitney test, ^b^independent sample t-test); comparison between ^*^ASD-diet and ASD-no diet, ^**^ASD group and TD group. ASD, autism spectrum disorder; ASD-diet, ASD children with gluten- and casein-free diet; ASD-no diet, ASD children without restricted diet; TD, typical development.

In Table 2, the comparison of genus and species relative abundance between nutritional status (normal weight vs. excess weight) and neurodevelopment (ASD vs. TD) groups was studied by two-way ANCOVA. Children in the All group (ASD + TD) with excess weight had a lower relative abundance of *Roseburia* than normal weight (*p* = 0.012). In addition, children with excess weight in the ASD group had a lower relative abundance of *Roseburia* (*p* = 0.005) and *Faecalibacterium prausnitzii* (*p* = 0.038) and a higher relative abundance of *Eubacterium ventriosum* (*p* = 0.019) and *Flavonifractor plautii* (*p* = 0.043) than those with excess weight in the TD group.

**Table 2:** Comparison of gut bacterial genus and species between nutritional status groups (normal weight vs. excess weight) and neurodevelopment groups (autism spectrum disorder vs. typical development) by two-way ANCOVA.

**Gut bacteria**	**ASD group**		**TD group**		**All (ASD** + **TD)**		**Pair comparisons**				
	**Normal weight (*n =* 20)**	**Excess weight (OW + OB) (*n =* 9)**	**Normal weight (*n =* 19)**	**Excess weight (OW + OB) (*n =* 9)**	**Normal weight (*n =* 39)**	**Excess weight (OW + OB) (*n =* 18)**	***P^a^***	***P^b^***	***P^c^***	***P^d^***	***P^e^***
**Genus**											
*Akkermansia*	1.12 ± 1.76	2.52 ± 3.17	1.73 ± 2.54	0.86 ± 0.90	1.42 ± 2.17	1.69 ± 2.42	0.282	0.812	0.518	0.956	0.295
*Alistipes*	3.69 ± 2.85	4.35 ± 4.03	4.84 ± 3.12	3.16 ± 2.17	4.25 ± 3.00	3.76 ± 3.20	0.215	0.172	0.997	0.905	0.051
*Bacteroides*	21.71 ± 10.19	24.86 ± 10.30	25.72 ± 10.75	21.78 ± 8.68	23.66 ± 10.52	23.32 ± 9.38	0.325	0.369	0.902	0.140	0.697
*Bifidobacterium*	2.36 ± 2.59	3.71 ± 4.13	3.73 ± 2.63	3.11 ± 1.66	3.03 ± 2.67	3.41 ± 3.07	0.565	0.986	0.655	0.668	0.871
*Blautia*	2.62 ± 1.36	2.40 ± 0.31	2.86 ± 1.42	2.21 ± 0.54	2.74 ± 1.37	2.30 ± 0.44	0.892	0.406	0.646	0.209	0.841
*Clostridium*	7.21 ± 3.68	6.29 ± 2.88	7.22 ± 2.99	6.22 ± 4.11	7.22 ± 3.32	6.25 ± 3.44	0.287	0.275	0.131	0.671	0.798
*Coprococcus*	0.88 ± 0.67	0.52 ± 0.49	0.76 ± 0.72	0.79 ± 0.65	0.82 ± 0.69	0.66 ± 0.58	0.188	0.509	0.585	0.645	0.211
*Dialister*	0.90 ± 1.49	1.01 ± 1.36	0.63 ± 0.72	0.77 ± 0.95	0.76 ± 1.16	0.89 ± 1.14	0.205	0.438	0.549	0.571	0.141
*Enterococcus*	0.46 ± 1.81	0.10 ± 0.12	0.43 ± 0.99	0.10 ± 0.13	0.45 ± 1.45	0.10 ± 0.12	0.927	0.235	0.462	0.251	0.901
*Eubacterium*	3.47 ± 2.32	2.49 ± 1.41	2.99 ± 1.89	3.24 ± 2.47	3.24 ± 2.10	2.87 ± 1.99	0.526	0.882	0.712	0.625	0.318
*Faecalibacterium*	14.84 ± 7.58	12.11 ± 4.38	15.14 ± 5.27	15.11 ± 5.01	14.99 ± 6.48	13.61 ± 4.82	0.596	0.983	0.706	0.260	0.197
*Lachnoclostridium*	0.86 ± 0.84	1.53 ± 1.15	0.83 ± 0.79	0.80 ± 0.52	0.84 ± 0.81	1.17 ± 0.94	0.192	0.913	0.371	0.420	0.567
*Lactobacillus*	0.94 ± 1.94	0.60 ± 0.58	0.46 ± 0.59	1.10 ± 2.07	0.71 ± 1.45	0.85 ± 1.50	0.456	0.461	0.928	0.172	0.913
*Prevotella*	11.52 ± 12.36	14.11 ± 11.79	6.67 ± 10.05	7.36 ± 7.45	9.16 ± 11.41	10.74 ± 10.18	0.402	0.505	0.287	0.372	0.392
*Pseudomonas*	0.52 ± 2.20	0.07 ± 0.04	0.07 ± 0.06	0.04 ± 0.03	0.30 ± 1.57	0.06 ± 0.04	0.658	0.367	0.353	0.654	0.924
*Roseburia*	2.73 ± 2.21	1.99 ± 1.09	2.82 ± 2.05	3.03 ± 1.69	2.77 ± 2.11	2.51 ± 1.48	0.965	0.560	0.671	**0.005**	**0.012**
*Ruminococcus*	2.37 ± 1.44	2.87 ± 1.68	3.01 ± 1.46	2.38 ± 1.00	2.68 ± 1.47	2.62 ± 1.36	0.550	0.434	0.932	0.193	0.912
*Streptococcus*	1.56 ± 4.72	0.83 ± 0.90	1.76 ± 1.94	1.04 ± 1.00	1.66 ± 3.60	0.94 ± 0.93	0.773	0.831	0.721	0.708	0.709
*Sutterella*	1.27 ± 1.48	1.57 ± 2.41	1.37 ± 1.61	2.66 ± 1.84	1.32 ± 1.53	2.12 ± 2.15	0.732	0.306	0.750	0.333	0.745
*Turicibacter*	0.81 ± 1.09	0.51 ± 0.74	0.61 ± 0.66	0.44 ± 0.46	0.71 ± 0.90	0.48 ± 0.60	0.593	0.799	0.568	0.422	0.750
**Species**											
*Akkermansia muciniphila*	0.97 ± 1.30	3.08 ± 3.82	2.09 ± 3.02	1.03 ± 1.14	1.51 ± 2.34	2.06 ± 2.93	0.184	0.619	0.517	0.634	0.261
*Bacteroides frágilis*	0.96 ± 2.38	0.45 ± 0.64	1.37 ±1.48	0.86 ± 0.85	1.16 ± 1.98	0.65 ± 0.76	0.804	0.727	0.681	0.530	0.646
*Bacteroides intestinalis*	0.02 ± 0.02	0.19 ± 0.41	0.80 ± 2.94	0.03 ± 0.03	0.34 ± 0.28	0.26 ± 0.42	0.435	0.274	0.421	0.381	0.872
*Bifidobacterium adolescentis*	0.64 ± 1.15	1.07 ± 2.25	0.54 ± 1.21	0.89 ± 0.90	0.59 ± 1.16	0.98 ± 1.66	0.612	0.719	0.545	0.575	0.539
*Bifidobacterium longum*	0.32 ± 0.45	1.34 ± 3.39	1.45 ± 2.13	0.71 ± 0.52	0.87 ± 1.60	1.02 ± 2.37	0.640	0.574	0.473	0.222	0.350
*Clostridium bartletti*	0.50 ± 0.57	0.56 ± 0.46	0.34 ± 0.26	0.26 ± 0.10	0.42 ± 0.44	0.411 ± 0.35	0.641	0.793	0.867	0.696	0.382
*Clostriduim glycolicum*	0.42 ± 0.34	0.63 ± 0.49	0.33 ± 0.34	0.26 ± 0.22	0.38 ± 0.34	0.45 ± 0.41	0.546	0.748	0.817	0.319	0.140
*Coprococcus comes*	0.33 ± 0.33	0.18 ± 0.18	0.28 ± 0.28	0.27 ± 0.19	0.311 ± 0.30	0.22 ± 0.19	0.511	0.786	0.496	0.304	0.699
*Eubacterium eligens*	1.76 ± 1.93	1.47 ± 1.06	1.09 ± 1.13	0.86 ± 0.70	1.43 ± 1.61	1.17 ± 0.93	0.410	0.658	0.755	0.780	0.418
*Eubacterium ventriosum*	7.91 ± 9.51	12.74 ± 4.95	13.60 ± 9.06	10.42 ± 8.61	10.68 ± 9.61	11.58 ± 6.92	0.064	0.415	0.414	**0.019**	0.719
*Faecalibacterium prausnitzii*	7.98 ± 10.60	0.13 ± 0.34	3.07 ± 6.55	5.93 ± 9.38	5.59 ± 9.10	3.03 ± 7.38	0.294	0.994	0.395	**0.038**	0.774
*Flavonifactor plautii*	0.32 ± 0.49	1.26 ± 1.18	0.89 ± 0.79	0.89 ± 1.14	0.60 ± 0.70	1.07 ± 1.14	0.223	0.295	0.846	**0.043**	0.915
*Lactobacillus reuteri*	0.11 ± 0.27	0.50 ± 0.59	0.30 ± 0.45	0.30 ± 0.35	0.20 ± 0.38	0.40 ± 0.48	0.696	0.773	0.624	0.464	0.502
*Lactobacillus salivarius*	0.39 ± 1.43	0.01 ± 0.04	0.02 ± 0.79	0.06 ± 0.09	0.21 ± 1.03	0.03 ± 0.07	0.637	0.422	0.966	0.218	0.847
*Roseburia hominis*	0.27 ± 0.38	0.21 ± 0.26	0.51 ± 1.62	0.45 ± 0.62	0.38 ± 1.16	0.33 ± 0.48	0.944	0.756	0.867	0.144	0.141
*Trabulsiella odonototermitis*	0.50 ± 1.21	0.45 ± 0.63	0.60 ± 0.71	0.28 ± 0.46	0.55 ± 0.99	0.36 ± 0.54	0.541	0.355	0.287	0.864	0.723

The results are expressed as means ± SD. Statistically significant differences (indicated in bold, *p* < 0.05; adjusted for age, birth weight, GCFD/not restricted diet): (a) Two-way ANCOVA, comparisons between ^a^normal and excess weight in the ASD group, ^b^normal and excess weight in the TD group, ^c^normal weight in ASD and TD, ^d^excess weight in ASD and TD; (b) one-way ANCOVA, comparison between ^e^normal weight and excess weight in all participants. ASD, autism spectrum disorder; TD, typical development; OW, overweight; OB, obesity.

**Figure 2:**
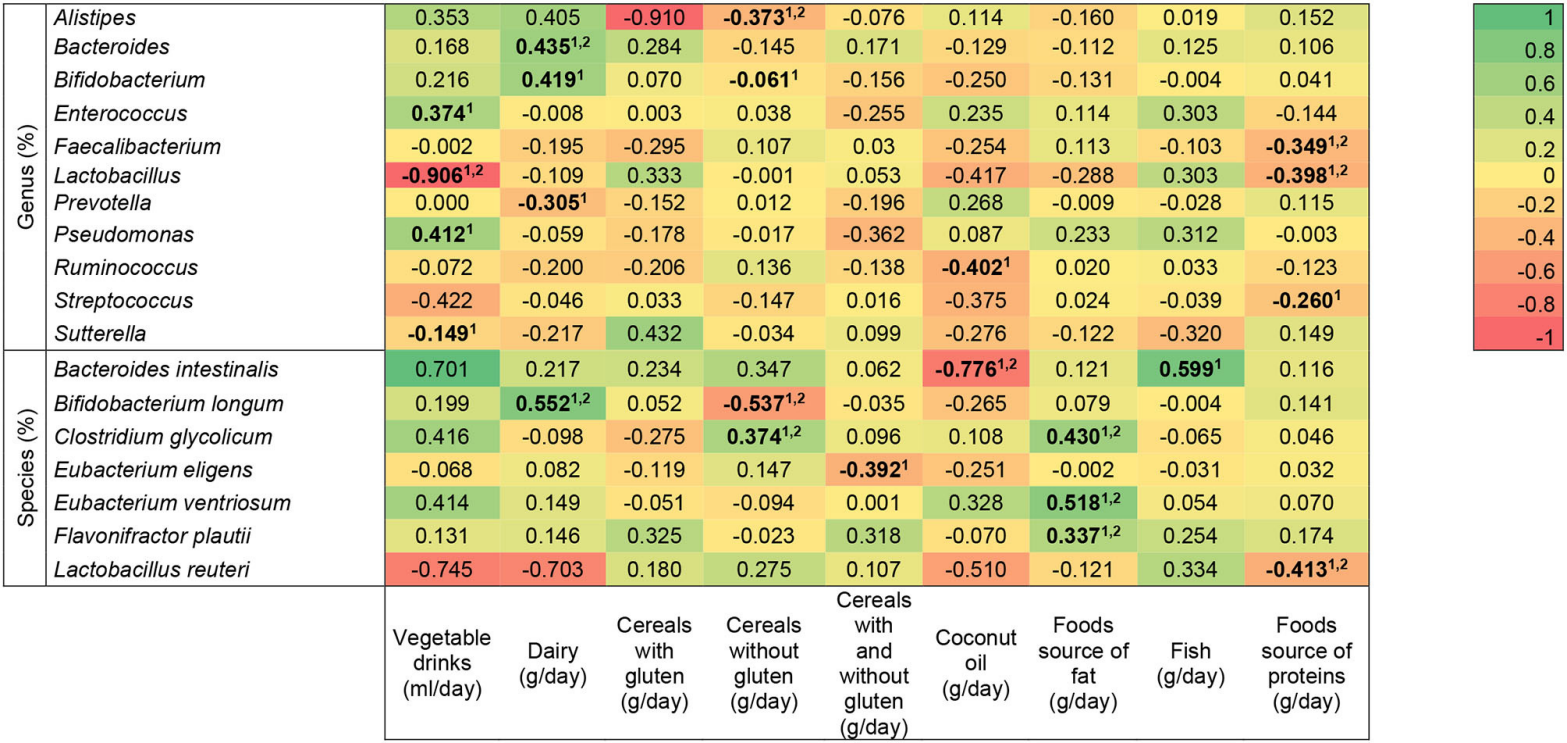
Heat map of the association between bacterial genera and species and dietary intake in children with autism spectrum disorder and neurotypical. Statistically significant differences (indicated in bold): *p* < 0.05 (linear regression model ^1^unadjusted, and ^2^adjusted for age, GCFD/not restricted diet, autism spectrum disorder/typical development). The R-values presented correspond to the adjusted models.

The relationship between the relative abundances of bacterial genus and species and children's dietary intake is shown in Figure 2. For the analysis, we applied unadjusted and adjusted (for age, GCFD/no diet and ASD/TD) models. Vegetable drink intake had an association with *Enterococcus* (*n* = 10; unadjusted model: *r* = 0.640, *p* = 0.046), *Pseudomonas* (*n* = 9; unadjusted: *r* = 0.671, *p* = 0.048), and *Sutterella* (unadjusted: *n* = 12, *r* = −0.692, *p* = 0.013), which disappeared after adjustment for covariates; and a relationship with *Lactobacillus* was independent of age, GCFD/no diet, and ASD/TD (*n* = 13; unadjusted: *r* = −0.683, *p* = 0.010; adjusted: *r* = −0.906, *p* = 0.009). Dairy intake (*n* = 43) had an association with *Bacteroides* (unadjusted: *r* = 0.353, *p* = 0.020; adjusted: *r* = 0.435, *p* = 0.048) and *Bifidobacterium longum* (unadjusted: *r* = 0.503, *p* = 0.001; adjusted: *r* = 0.552, *p* = 0.009) that remained when the covariates were applied; it also had a relationship with *Bifidobacterium* (unadjusted: *n* = 43, *r* = 0.320, *p* = 0.036) and *Prevotella* (unadjusted: *n* = 43, *r* = −0.380, *p* = 0.012) which did not hold after adjustment. Cereals with gluten did not show a significant correlation with bacteria genus or species. However, cereals without gluten (*n* = 58) had a relationship with *Alistipes* (unadjusted: *r* = −0.336, *p* = 0.015; adjusted: *r* = −0.373, *p* = 0.024), *Bifidobacterium longum* (unadjusted: *r* = −0.418, *p* = 0.003; adjusted: *r* = −0.537, *p* = 0.006), and *Clostridium glycolicum* (*n* = 58; unadjusted: *r* = 0.364, *p* = 0.009; adjusted: *r* = 0.374, *p* = 0.021), independent of confounding factors. They also had an association with *Bifidobacterium* (*n* = 56; unadjusted: *r* = −0.290, *p* = 0.041), which disappeared after adjustment for covariates. In addition, the association between cereals with and without gluten intake and *Eubacterium eligens* (*n* = 58; unadjusted: *r* = −0.290, *p* = 0.027) and coconut oil intake with *Ruminococcus* (*n* = 58; unadjusted: *r* = −0.532, *p* = 0.011) disappeared after adjustment. The relationship between coconut oil intake and *Bacteroides intestinalis* (*n* = 58 unadjusted: *r* = −0.891, *p* = 0.001; adjusted: *r* = −0.776, *p* = 0.021) and food source of fat (*n* = 58) with *Clostridium glycolicum* (unadjusted: *r* = 0.442, *p* = 0.001; adjusted: *r* = −0.430, *p* = 0.001)*, Eubacterium ventriosum* (unadjusted: *r* = 0.356, *p* = 0.008; adjusted: *r* = 0.518, *p* = 0.004), *and Flavonifractor plautii* (unadjusted: *r* = 0.325, *p* = 0.026; adjusted: *r* = 0.337, *p* = 0.026) was independent of confounding factors. Fish intake was associated with *Bacteroides intestinalis* (*n* = 28; unadjusted: *r* = 0.582, *p* = 0.023) only in the model without adjustment. Food source of proteins had a relationship with *Faecalibacterium* (*n* = 58; unadjusted: *r* = −0.358, *p* = 0.006; adjusted: *r* = −0.349, *p* = 0.007), *Lactobacillus* (*n* = 49; unadjusted: *r* = −0.365, *p* = 0.010; adjusted: *r* = −0.398, *p* = 0.005), and *Lactobacillus reuteri* (*n* = 58; unadjusted: *r* = −0.363, *p* = 0.044; adjusted: *r* = −0.413, *p* = 0.027) in unadjusted and adjusted models, but the association with *Streptococcus* disappeared after considering potential confounders (*n* = 56; unadjusted: *r* = −0.287, *p* = 0.032).

**Figure 3:**
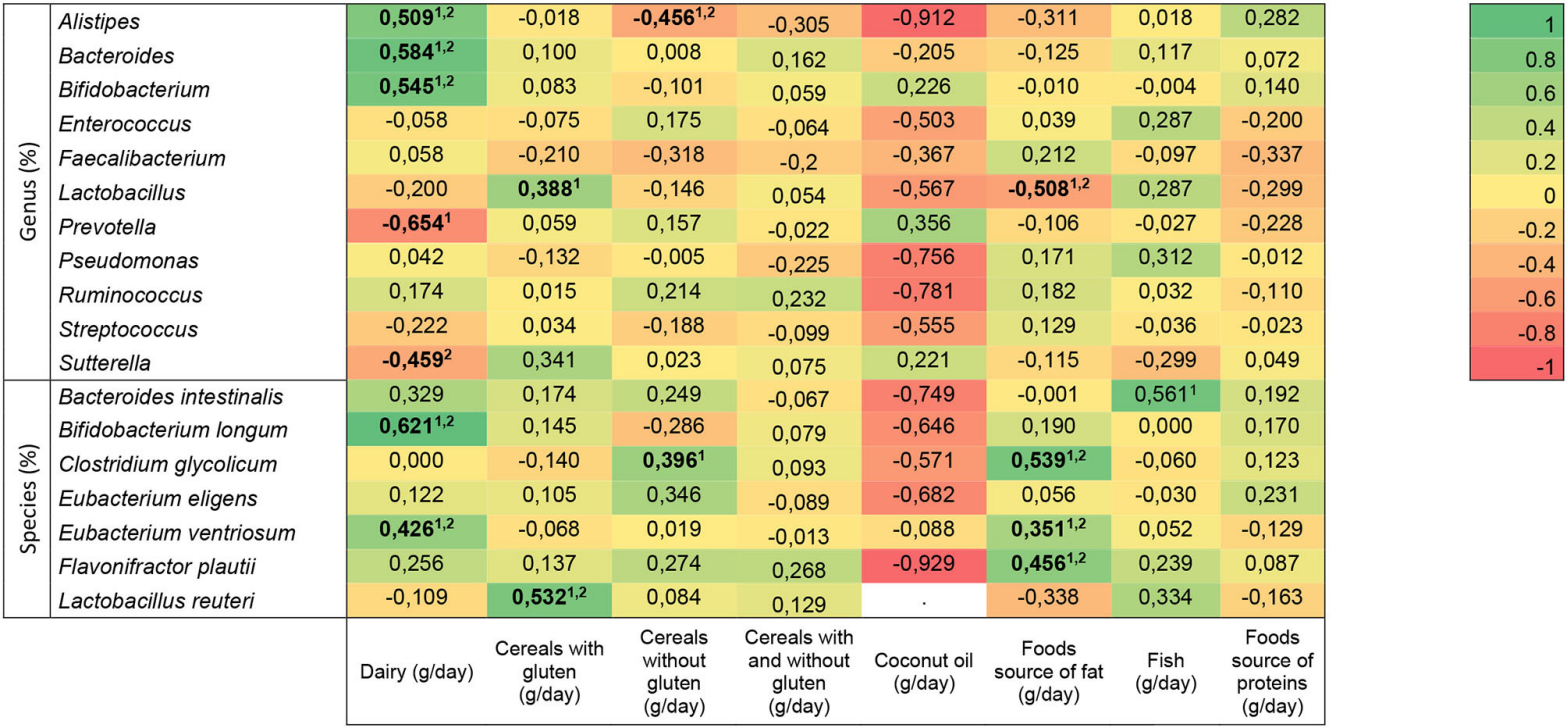
Heat map of the association between bacterial genera and species and dietary intake in neurotypical children. Statistically significant differences (indicated in bold): *p* < 0.05 (linear regression model ^1^unadjusted, and ^2^djusted for age). The R-values presented correspond to the adjusted models.

The association between bacteria genus and species and dietary intake in children with typical development is presented in Figure 3, where unadjusted and age-adjusted models were used. Dairy intake (*n* = 43) had an association with *Alistipes* (unadjusted: *r* = 0.427, *p* = 0.026; adjusted: *r* = 0.509, *p* = 0.022), *Bacteroides* (unadjusted: *r* = 0.498, *p* = 0.008; adjusted: *r* = 0.584, *p* = 0.007)*, Bifidobacterium ventrosum* (unadjusted: *r* = 0.388, *p* = 0.046; adjusted: *r* = 0.545, *p* = 0.013), *Bifidobacterium longum* (unadjusted: *r* = 0.544, *p* = 0.004; adjusted: *r* = 0.621, *p* = 0.007), and *Eubacterium ventrosum* (unadjusted: *r* = 0.541, *p* = 0.004; adjusted: *r* = 0.426, *p* = 0.021), independent of the age, but the association with *Prevotella* (unadjusted: *r* =−0.547, *p* = 0.003) disappeared after adjustment, and the relationship with *Sutterella* (adjusted: *r* =−0.459, *p* = 0.045) only appeared when the covariate was considered. Cereals with gluten had a relationship with *Lactobacillus* (unadjusted: *n* = 49, *r* = 0.525, *p* = 0.007) that disappeared after adjusting, and with *Lactobacillus reuteri*, it was remained independent (unadjusted: *n* = 58, *r* = 0.579, *p* = 0.015, and adjusted: *r* = 0.532, *p* = 0.047). Cereals without gluten intake had an association with *Alistipes*, independent of age (unadjusted: *n* = 58, *r* =−0.439, *p* = 0.028; adjusted: *r* =−0.456, *p* = 0.028), but the association with *Clostridium glycolicum* disappeared when considering the confounder (unadjusted: *n* = 58, *r* = 0.402, *p* = 0.047). Food source of fat had an association with *Lactobacillus* (*n* = 49, unadjusted: *r* =−0.563, *p* = 0.003; adjusted: *r* =−0.508, *p* = 0.004), *Clostridium glycolicum* (*n* = 58; unadjusted: *r* = 0.546, *p* = 0.003; adjusted: *r* = 0.539, *p* = 0.004)*, Eubacterium ventriosum* (*n* = 58; unadjusted: *r* = 0.407, *p* = 0.035; adjusted: *r* = 0.351, *p* = 0.050), *and Flavonifractor plautii* (*n* = 58; unadjusted: *r* = 0.487, *p* = 0.016; adjusted: *r* = 0.456, *p* = 0.021), which remained after adjusting. Fish had an association with *Bacteroides intestinalis* (unadjusted: *n* = 58, *r* = 0.555, *p* = 0.039), but it disappeared after considering the age. No significant associations were found between bacteria genera or species and cereals with and without gluten, coconut oil, and food source of proteins.

**Figure 4:**
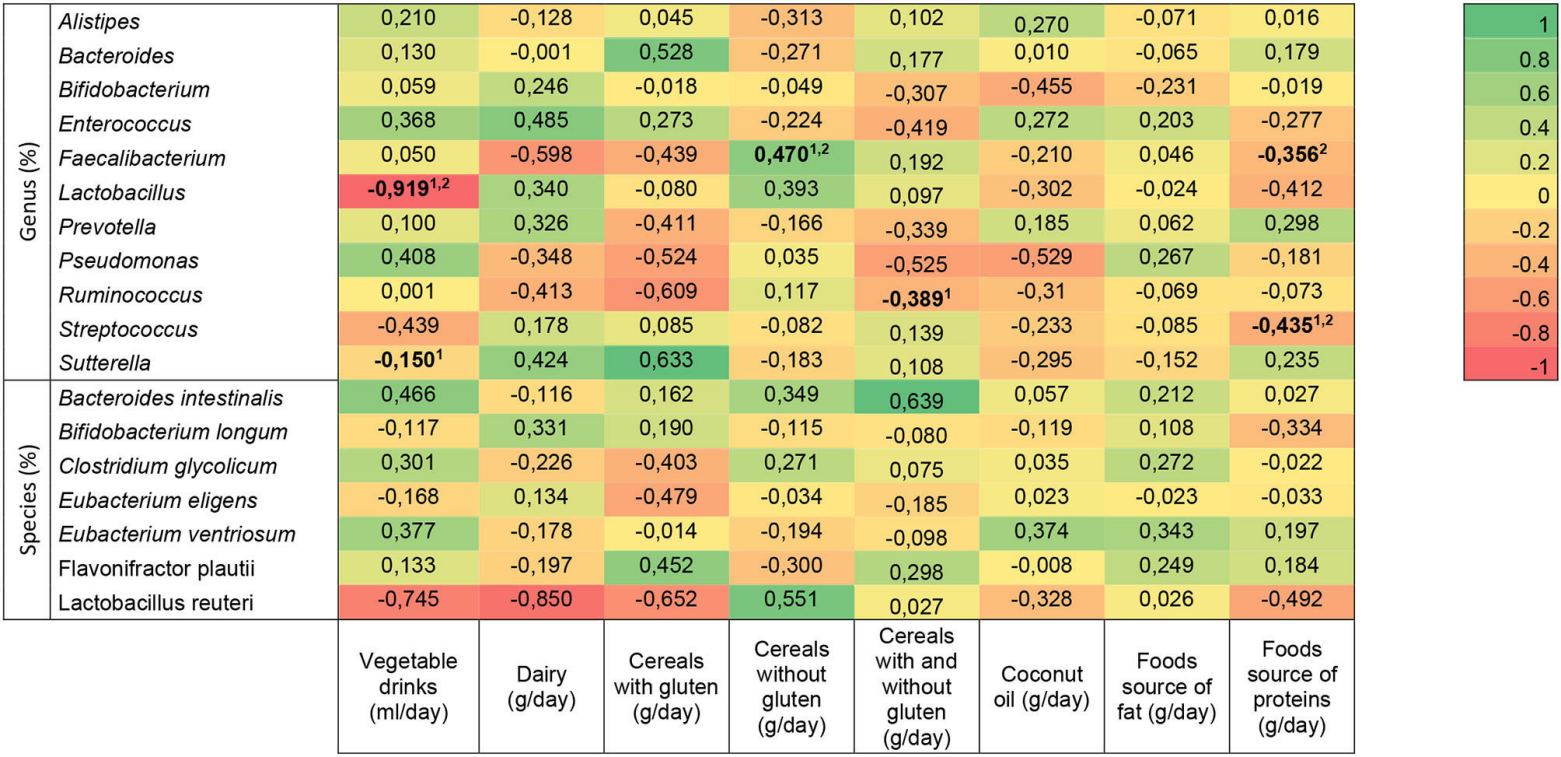
Heat map of the association between bacterial genera and species and dietary intake in autism spectrum disorder children. Statistically significant differences (indicated in bold): *p* < 0.05 (linear regression model ^1^unadjusted, and ^2^adjusted for age, gluten- and casein-free diet/no diet). The *R*-values presented correspond to the adjusted models.

The association between dietary intake and relative abundance of bacteria genus and species in children with ASD is shown in Figure 4. The analysis was unadjusted and adjusted for age and gluten- and casein-free diet/no diet. Vegetable drinks were associated with *Lactobacillus* in both models (*n* = 49, unadjusted: *r* =−0.620, *p* = 0.042; adjusted: *r* =−0.919, *p* = 0.034), but they were associated with *Sutterella* only without considering confounding factors (*n* = 49; unadjusted: *r* =−0.645, *p* = 0.032). Intake of dairy, cereals with gluten, coconut oil, and food source of fat did not show a significant correlation with gut bacteria. Cereals without gluten had a correlation with *Faecalibacterium* which was kept after adjustment (*n* = 58; unadjusted: *r* = 0.482, *p* = 0.011; adjusted: *r* = 0.470, *p* = 0.040). Cereals with and without gluten had an association with *Ruminococcus* (*n* = 58; unadjusted: *r* =−0.388, *p* = 0.034), but it disappeared when potential confounders were considered. The association between food source of proteins and *Faecalibacterium* was observed only when considering the covariates (*n* = 58; adjusted: *r* =−0.356, *p* = 0.049); with *Streptococcus*, the relationship was found in unadjusted and adjusted models (*n* = 58; unadjusted: *r* =−0.437, *p* = 0.020; adjusted: *r* =−0.435, *p* = 0.023).

Table 3 shows the comparison of gut bacterial genus and species between dietary intake groups, categorized by the 50th percentile, in children with TD and ASD without a GCFD by means of two-way ANOVA. Children with ASD-no diet and a dairy intake of >334.3 g/day had a higher relative abundance of *Lactobacillus* (*p* = 0.025) and *Streptococcus* (*p* = 0.003) than those with an intake of ≤334.3 g/day. In the TD group with a dairy intake of >334.3 g/day, a greater amount of *Alistipes* (*p* = 0.013) and *Eubacterium ventriosum* (*p* = 0.024) was found as compared with children with a lower dairy intake. When gut bacteria were compared between TD and ASD-no diet children in the dairy intake of >334.3 g/day group, we observed a lower relative abundance of *Faecalibacterium* (*p* = 0.005) and a higher one of *Lactobacillus* (*p* = 0.012) and *Streptococcus* (*p* = 0.009) in ASD-no diet children. In relation to cereals with gluten*, Faecalibacterium* was higher (*p* = 0.042) in ASD-no diet children with an intake of >198.35 g/day than TD with the same intake. ASD-no diet children with an intake of cereals without gluten ≤7.12 g/day had higher *Flavonifractor plautii* (*p* = 0.013) than those with an intake of >7.12 g/day. In addition, in the group with an intake of cereal without gluten >7.12 g/day, *Flavonifractor plautii* (*p* = 0.014) and *Eubacterium ventrosum* (*p* = 0.017) were higher in TD children than in ASD-no diet. Regarding food source of fat, when the intake was >25 g/day, the abundance of *Faecalibacterium* was higher in TD children than in ASD-no diet (*p* = 0.031).

**Table 3:** Comparison of gut bacterial genus and species between dietary intake groups (categorized by the 50th percentile) in children with typical development and autism spectrum disorder without diet by means of two-way ANOVA.

**Gut bacteria**	**ASD-no diet group**		**TD group**		**ASD-no diet group**		**TD group**		**ASD-no diet group**		**TD group**		**ASD-no diet group**		**TD group**	
	**Dairy (g/day)**				**Cereals with gluten (g/day)**				**Cereals without gluten (g/day)**				**Foods source of fat (g/day)**			
	≤**334.3 (*****n** =* **11)**	>**334.3 (*****n** =* **3)**	≤**334.3 (*****n** =* **10)**	>**334.3 (*****n** =* **18)**	≤**198.3 (*****n** =* **9)**	>**198.3 (*****n** =* **5)**	≤**198.3 (*****n** =* **13)**	>**198.3 (*****n** =* **15)**	≤**7.1 (*****n** =* **8)**	>**7.1 (*****n** =* **6)**	≤**7.1 (*****n** =* **13)**	>**7.1 (*****n** =* **15)**	≤**25.0 (*****n***=**7)**	>**25.0 (*****n** =* **7)**	≤**25.0 (*****n** =* **14)**	>**25.0 (*****n** =* **14)**
**Genus**																
*Alistipes*	4.07 ± 2.81	5.67 ± 6.52	**2.70** **±** **1.87**^**b**^	**5.19** **±** **3.05**^**b**^	4.19 ± 3.00	4.81 ± 4.91	4.63 ± 2.50	4.01 ± 3.30	4.28 ± 4.00	4.65 ± 3.22	5.06 ± 3.13	2.93 ± 1.96	4.98 ± 4.11	3.85 ± 3.26	4.85 ± 3.00	3.75 ± 2.84
*Bacteroides*	21.57 ± 8.88	20.56 ± 1.23	22.24 ± 11.41	25.68 ± 9.48	20.74 ± 7.52	22.45 ± 9.12	22.97 ± 6.37	25.74 ± 12.64	3.91 ± 3.40	4.79 ± 3.95	5.06 ± 3.07	3.64 ± 2.71	21.34 ± 6.80	21.36 ± 9.28	26.76 ± 11.47	22.14 ± 8.40
*Bifidobacterium*	4.58 ± 3.99	2.87 ± 1.48	2.26 ± 1.59	4.24 ± 2.44	5.34 ± 4.04	2.19 ± 1.46	4.06 ± 2.15	3.08 ± 2.49	3.82 ± 3.60	4.74 ± 3.92	4.16 ± 2.57	2.99 ± 2.08	3.66 ± 3.21	4.77 ± 4.18	3.36 ± 2.16	3.71 ± 2.59
*Faecalibacterium*	13.12 ± 4.04	**6.55** **±** **2.55**^**c**^	14.14 ± 5.02	**15.68** **±** **5.19**^**c**^	12.88 ± 4.42	**9.60** **±** **4.67**^**c**^	15.58 ± 4.12	**14.74** **±** **5.93**^**c**^	12.27 ± 4.83	10.96 ± 4.67	16.20 ± 6.15	14.20 ± 3.95	12.08 ± 4.85	**11.34** **±** **4.74**^**c**^	13.97 ± 5.57	**16.29** **±** **4.46**^**c**^
*Lactobacillus*	**0.50** **±** **0.41**^**a**^	**2.96** **±** **5.10**^**a, c**^	1.24 ± 2.00	**0.35** **±** **0.34**^**c**^	0.51 ± 0.44	1.95 ± 3.86	0.78 ± 1.76	0.57 ± 0.63	0.40 ± 0.41	1.86 ± 3.45	0.94 ± 1.74	0.43 ± 0.61	0.44 ± 0.43	1.61 ± 3.21	0.99 ± 1.73	0.35 ± 0.35
*Streptococcus*	**0.86** **±** **0.89**^**a**^	**7.43** **±** **12.22**^**a, c**^	0.78 ± 0.53	**1.95** **±** **2.00**^**c**^	1.02 ± 0.92	4.53 ± 9.51	2.18 ± 2.07	0.97 ± 1.13	4.26 ± 8.50	0.78 ± 0.91	1.97 ± 2.19	1.15 ± 1.10	0.81 ± 0.96	3.73 ± 7.88	1.13 ± 1.53	1.93 ± 1.85
*Suterella*	2.33 ± 2.14	1.38 ± 1.29	2.36 ± 1.73	1.46 ± 1.75	1.73 ± 1.72	2.85 ± 2.43	1.88 ± 2.05	1.69 ± 1.54	1.92 ± 2.25	2.40 ± 1.73	1.63 ± 1.72	1.91 ± 1.85	1.43 ± 1.36	2.82 ± 2.36	1.47 ± 1.79	2.09 ± 1.74
**Species**																
*Bifidobacterium longum*	1.29 ± 3.01	0.47 ± 0.15	0.47 ± 0.50	1.62 ± 2.12	1.51 ± 3.32	0.39 ± 0.23	1.22 ± 1.93	1.20 ± 1.75	0.34 ± 0.26	2.14 ± 4.02	1.37 ± 1.81	1.07 ± 1.84	0.45 ± 0.25	1.78 ± 3.78	0.68 ± 0.52	1.74 ± 2.42
*Clostridium glycolicum*	0.49 ± 0.50	0.78 ± 0.45	0.25 ± 0.21	0.34 ± 0.35	0.63 ± 0.56	0.41 ± 0.31	0.37 ± 0.38	0.26 ± 0.22	0.43 ± 0.26	0.71 ± 0.68	0.22 ± 0.21	0.39 ± 0.36	0.47 ± 0.37	0.64 ± 0.60	0.23 ± 0.22	0.39 ± 0.36
*Eubacterium ventriosum*	9.57 ± 8.76	5.04 ± 4.80	**5.76** **±** **7.79**^**b**^	**16.36** **±** **7.13**^**b**^	9.60 ± 8.82	6.82 ± 7.31	14.95 ± 8.35	10.52 ± 9.10	10.76 ± 8.64	**5.73** **±** **7.11**^**c**^	11.50 ± 10.21	**13.51** **±** **7.81**^**c**^	8.20 ± 9.63	9.01 ± 7.10	10.83 ± 9.08	14.33 ± 8.66
*Lactobacillus reuteri*	0.22 ± 0.47	0.01 ± 0.03	0.14 ± 0.33	0.38 ± 0.43	0.25 ± 0.52	0.04 ± 0.08	0.27 ± 0.32	0.32 ± 0.49	0.31 ± 0.53	0.00 ± 0.01	0.35 ± 0.50	0.25 ± 0.33	0.24 ± 0.57	0.12 ± 0.23	0.35 ± 0.45	0.24 ± 0.38
*Flavonifactor plautii*	0.73 ± 1.03	0.86 ± 1.24	0.38 ± 0.49	1.18 ± 0.95	0.67 ± 1.10	0.91 ± 0.97	1.11 ± 0.91	0.70 ± 0.86	**1.27** **±** **1.12**^**a**^	**0.08** **±** **0.11**^**a**^	0.71 ± 0.89	1.05 ± 0.90	0.68 ± 0.84	0.83 ± 1.25	0.69 ± 0.56	1.10 ± 1.12

The results expressed as means ± SD. Statistically significant differences (indicated in bold, *p* < 0.05): Two-way ANOVA, comparisons between ^a^lower and higher dietary intake groups in the ASD-no diet children; ^b^lower and higher dietary intake groups in TD children; ^c^ASD-no diet and TD children in the higher dietary intake group. ASD-no diet, children with autism spectrum disorder without a restricted diet; TD, typical development.

Table 4 shows the comparison of gut bacterial genus and species between dietary intake groups categorized by the 50th percentile in children with ASD and a GCFD. *Alistipes* was higher (*p* = 0.002), and *Lactobacillus* was lower (*p* = 0.004) in the >309.5 ml/day vegetable drink intake group than in children with an intake of ≤309.5 ml/day. Higher levels of *Faecalibacterium* (*p* = 0.045) were found in individuals who consume >102.46 g/day of cereals without gluten compared with those with lower intake. No significant difference was found when comparing gut bacteria between the higher and the lower intake of food source of proteins.

**Table 4:** Comparison of gut bacterial genus and species between dietary intake groups (categorized by the 50th percentile) in children with autism spectrum disorder and a gluten- and casein-free diet.

**Gut bacteria**	**Vegetable drinks (ml/day)**			**Cereals without gluten (g/day)**			**Foods source of proteins (g/day)**		
	** ≤309.5 (*n =* 8)**	**>309.5 (*n =* 8)**	***p***	** ≤102.4 (*n =* 8)**	**>102.4 (*n =* 8)**	***p***	** ≤183.6 (*n =* 8)**	**>183.6 (*n =* 8)**	***p***
**Genus**									
*Alistipes*	1.56 ± 1.11	5.64 ± 2.51	**0.002** ^**a**^	2.69 ± 2.46	4.51 ± 3.01	0.327^a^	3.60 ± 3.41	3.60 ± 2.32	0.860^a^
*Bacteroides*	22.72 ± 8.34	25.24 ± 14.77	0.111^a^	24.13 ± 12.24	23.82 ± 11.91	0.559^a^	22.44 ± 5.66	25.51 ± 15.94	0.826^a^
*Bifidobacterium*	2.15 ± 2.28	0.73 ± 0.79	0.156^b^	2.01 ± 2.35	0.86 ± 0.84	0.275^b^	1.36 ± 0.91	1.52 ± 2.48	0.936^b^
*Faecalibacterium*	14.90 ± 4.60	17.77 ± 10.02	0.458^a^	12.59 ± 6.12	20.08 ± 7.54	**0.045** ^a^	16.72 ± 3.84	15.95 ± 10.55	0.862^b^
*Lactobacillus*	1.06 ± 0.67	0.22 ± 0.22	**0.004** ^b^	0.64 ± 0.75	0.64 ± 0.58	0.925^b^	0.94 ± 0.56	0.34 ± 0.62	0.062^b^
*Streptococcus*	0.60 ± 0.31	0.38 ± 0.33	0.476^b^	0.47 ± 0.38	0.51 ± 0.30	0.476^b^	0.60 ± 0.32	0.38 ± 0.32	0.090^b^
*Suterella*	1.12 ± 1.61	0.11 ± 0.14	0.108^b^	1.11 ± 1.61	0.12 ± 0.15	0.124^b^	0.89 ± 1.60	0.34 ± 0.68	0.372^b^
**Species**									
*Bifidobacterium longum*	0.31 ± 0.67	0.15 ± 0.23	0.923^a^	0.37 ± 0.68	0.09 ± 0.12	0.511^a^	0.16 ± 0.23	0.30 ± 0.68	0.772^a^
*Clostridium glycolicum*	0.39 ± 0.27	0.45 ± 0.30	0.937^b^	0.30 ± 0.23	0.54 ± 0.28	0.147^b^	0.51 ± 0.29	0.34 ± 0.26	0.310^b^
*Eubacterium ventriosum*	9.08 ± 8.10	12.46 ± 10.46	0.330^b^	9.10 ± 7.70	12.44 ± 10.76	0.401^b^	10.42 ± 8.91	11.11 ± 10.09	0.943^b^
*Lactobacillus reuteri*	0.45 ± 0.56	0.13 ± 0.20	0.169^b^	0.21 ± 0.27	0.37 ± 0.57	0.430^b^	0.49 ± 0.54	0.09 ± 0.18	0.059^b^
*Flavonifactor plautii*	0.72 ± 0.88	1.18 ± 2.78	0.311^b^	1.44 ± 2.72	0.46 ± 0.82	0.511^b^	1.45 ± 2.78	0.46 ± 0.59	0.251^b^

The results are expressed as means ± SD. Statistically significant differences (indicated in bold): *p* < 0.05 (^a^t-test, ^b^Mann–Whitney test).

## Discussion

In this study, differences were observed in the composition of fecal bacteria as per nutritional status and dietary intake in ASD and TD children. Several studies have shown different gut bacteria compositions in children with ASD as compared with TD (25). In contrast, we have not found significant differences at the genus level, but at the species level, we observed lower abundances of *Bifidobacterium longum* and higher abundances of *Clostridium glycolicum* in ASD than in the TD group. Wang et al. (26) reported lower levels of *Bifidobacterium spp*. and *Akkermansia muciniphila* in children with autism compared with TD ones, but in our study, no significant difference was observed in the abundance of this mucolytic bacterium. Our findings are in line with previous studies that found decreased *Bifidobacterium* spp. and elevated *Clostridium* spp. in ASD as compared with controls and suggested that the latter bacterium is a determinant of the risk of autism (12, 27, 28). Research conducted in 1998 hypothesized that ASD could be due to a dysbiosis context with colonization by *Clostridium tetani* and to its neurotoxic effects in neurons producing gamma-aminobutyric acid (inhibitory neurotransmitter of the central nervous system) (29, 30). Another study shows a positive correlation between *Clostridium* cluster XVIII and gastrointestinal symptoms such as constipation in autistic and neurotypical subjects (27, 31).

Interestingly, in the ASD-diet group, *Bifidobacterium* (*B.)* were significantly lower compared with the ASD-no diet group, and this bacteria had a positive association with dairy intake and a negative association with cereals without gluten in the All (ASD + TD) group. A greater abundance of *Bifidobacterium* has been described as having beneficial effects on health since it inhibits pathogen growth by releasing bacteriocins (32, 33), and an increase in *B. longum* can mitigate depression in patients with irritable bowel syndrome through changes in the brain areas involved in mood regulation (34).

In line with our results, a study in adults shows a significant association between gluten-free diet and a reduced relative abundance of *Bifidobacterium* and *Lactobacillus (L.)* (35). Importantly, both genera synthesize short-chain fatty acids, that interact with receptors in the gut mucosa and contribute to mucus maintenance, have an antimicrobial effect on pathogens and can reverse leaky gut disorders (36). Therefore, the assumption is that greater abundances of *Lactobacillus* in the gut are associated with a higher intake of dairy (37). In the present study, we also observed that children with ASD-no diet and a dairy intake of >334.3 g/day had a higher mean of *Lactobacillus* than those with a lower intake. On the contrary, the ASD-diet group with an intake of >309.5 ml/day of vegetable drinks had a lower abundance of this genus than children with an intake of ≤309.5 ml/day. Considering all participants, the intake of vegetable drinks and food source of proteins had a negative association with *Lactobacillus* and *L. reuteri*, respectively. This last bacterial species was found to reverse social deficits in experimental animals with ASD (38).

Apart from that, vegetable drink intake also had a positive association with the facultative anaerobe *Enterococcus* and a negative association with *Sutterella* in all children only in the unadjusted model. In relation to this, Mangiola et al. (39) have reported a positive association between *Sutterella* genus and the development of autism in children. Nevertheless, an increase in *Enterococcus* has been detected in fecal samples from patients with diarrhea (40). In addition, a pro-inflammatory bacteria named *Alistipes* (41) was higher in ASD-diet children with an intake of >309.5 ml/day of vegetable drinks than those with a lower intake, but it was also higher in children with TD and a dairy intake of >334.3 g/day than in those with a dairy intake of ≤334.3 g/day, and a positive association between *Alistipes* and dairy intake was observed in this group. Additionally, in All and TD groups, this bacterium was inversely related to cereals without gluten. Another bacterium with anti-inflammatory properties called *Prevotella* (42) had a negative association (only in the unadjusted model) with dairy intake considering All and TD children.

In relation to nutritional status, participants in the All group with excess weight had a lower relative abundance of a beneficial butyrate-producing bacterium called *Roseburia (R.)* (43) than the normal weight ones, and *Roseburia* and *R. hominis* were higher in the ASD-diet as compared with the ASD-no diet group. In addition, children with excess weight in the ASD group had a significantly lower relative abundance of *Roseburia* and *Faecalibacterium prausnitzii* and a higher relative abundance of *Eubacterium ventriosum* and *Flavonifractor plautii* than those with excess weight in the TD group. A study performed in Japan showed that *E. ventriosum* was significantly associated with obese subjects (44). Moreover, a significantly reduced abundance of this species was described in people with colorectal cancer and could be considered a risk biomarker for the illness (45). On the other hand, *Faecalibacterium prausnitzii* is responsible for degrading mucin-producing butyrate and peptides that inhibit the NF-kB pathway in intestinal epithelial cells with an anti-inflammatory effect, and it has been associated with a reduced abundance in obesity (46, 47).

It has recently been reported that eating bread made from transgenic low-gliadin wheat produces a significantly higher abundance of *Faecalibacterium* and *Roseburia* genera with potentially beneficial changes in the composition of the intestinal microbiota, due to the increase in butyrate, which maintains good gut permeability (48). In our study, considering the ASD group, cereals without gluten also had a positive correlation with *Faecalibacterium*. Additionally, we observed that the ASD-diet group had a significantly higher level of *Faecalibacterium* in individuals who consume >102.46 g/day of cereals without gluten as compared with those with lower intake. It is worth mentioning that Jiang et al. (49) observed a negative correlation between *Faecalibacterium* abundances and the severity of depressive manifestation and overexpression of *Alistipes* in this psychiatric disorder.

In relation to cereal intake, other studies have shown that a high carbohydrate intake was associated with higher abundances of *Bifidobacterium* and *Lactobacillus* in fecal samples (50). In our study, the TD group precisely had a positive relationship between the intake of cereals with gluten and the abundances of *Lactobacillus* and *L. reuteri*. Apart from that, by comparison, children with TD and an intake of >7.12 g/day of cereals without gluten had higher abundances of *Flavonifractor (F.) plautii* than the ASD-no diet group with the same food intake, and in this last group, those with an intake of ≤7.12 g/day had more *F. plautii* than children with an intake of >7.12 g/day. Mikami et al. (51) have recently reported that an increased abundance of this bacterium has a beneficial effect as a modulator of gut inflammation, mediating IL-17 suppression in animals.

A number of studies have reported that an animal-based diet with high protein and fat intake seems to increase bile-tolerant bacteria called *Bacteroides* and could boost intestinal bowel disease risk (15, 52). Consistent with the said finding, we found that fish intake was positively associated with *Bacteroides intestinalis* in All and TD children (without adjustment for potential confounders). Even so, an investigation with mice fed with fish oil for 11 weeks described that, due to the interaction with the gut microbiota, there was less white adipose tissue inflammation and Toll-like receptor activation as compared with the lard diet (53). However, another research shows that in humans, salmon consumption has no effect on gut microbiota of pregnant women (54).

Regarding the intake of food source of fat, a comparison study showed that rats on a diet rich in coconut oil for 2 weeks had a lower abundance of *Ruminococcus flavecaciens* than those fed with soy oil (55). Along those lines, our study has found a negative association between coconut oil intake and *Ruminococcus* (unadjusted model) and *Bacteroides intestinalis* in the All group. In addition, a meta-analysis shows 11 trials with a higher abundance of *Ruminococcus* (involved in the fermentation of dietary fibers) in ASD children than in TD ones (25), but we have not found a significant difference in those groups.

In short, advances in the study of intestinal microbiota have led to new research, but the results have been disparate due to the complexity of the subject. A number of investigations show that gut microbiota may modulate brain function via metabolic and signaling pathways in charge of social cognition and emotional regulation (56). Our results show how gut microbiota composition is related to food consumption, nutritional status, and neurodevelopment. Based on these results and the possibility of further investigating the interaction between diet, gut microbiota, and autism through intervention studies, it would be possible to establish a clear relationship between specific bacteria profiles, food intake, and neurodevelopment. This could help establish preventive and treatment strategies for autism. Further studies focusing on an associated analysis of these topics in a large sample of children are needed to improve recommendations for this population.

## Study limitations

Research limitation is related to the small size of the sample, and the fact that the amount of dietary intake was estimated by the food frequency questionnaire instead of being accurately measured. However, these questionnaires are the most economical and validated method used worldwide, and we consider this study as an important input to the knowledge on this topic and for other types of research, since there are few studies that address the relationship of the intestinal microbiota with dietary intake and nutritional status in children with ASD.

## Conclusion

In this study, we observed differences in the composition of gut bacteria in children with autism spectrum disorder and typical development in only two species (*Bifidobacterium longum* and *Clostridium glycolicum*), but when we analyzed these two populations taking into account dietary intake and nutritional status, we were able to observe more differences. We found positive and negative associations between the intake of dairy, vegetable drinks, cereals with gluten and without gluten, food source of proteins, fish, food source of fat, and coconut oil, with the gut microbiota, independent of potential confounder variables such as age, being on a gluten- and casein-free diet, and neurodevelopment. Moreover, analyzing and comparing the higher and lower intake of these food groups allowed us to observe in greater depth how intake quantities are associated with higher or lower abundances of gut bacteria. Pending further studies, these results might be considered as a starting point for the nutritional treatment of ASD children.

## Data availability statement

The data presented in the study are deposited in the NCBI with links to BioProject accession number PRJNA988151 in the NCBI BioProject database (https://www.ncbi.nlm.nih.gov/bioproject/PRJNA988151).

## Ethics statement

The studies involving human participants were reviewed and approved by the Research Ethics Committee of the Universidad de la República's School of Nurition (CEIN). Written informed consent to participate in this study was provided by the participants' legal guardian/next of kin.

## Author contributions

PM: designed and conducted research, analyzed data, performed statistical analysis, wrote the manuscript, and had primary responsibility for final content. MG: designed research, analyzed data, performed statistical analysis, had primary responsibility for final content, and supervision and review. All authors contributed to the article and approved the submitted version.
